# The change of Barthel Index scores from the time of discharge until 3-month post-discharge among acute stroke patients in Malaysia: A random intercept model

**DOI:** 10.1371/journal.pone.0208594

**Published:** 2018-12-20

**Authors:** Kamarul Imran Musa, Thomas J. Keegan

**Affiliations:** 1 Department of Community Medicine, School of Medical Sciences, Universiti Sains Malaysia, Kbg Kerian, Kelantan, Malaysia; 2 The Centre for Health Informatics, Computing, and Statistics (CHICAS), Lancaster Medical School, Lancaster University, Lancaster, United Kingdom; Taipei Veterans General Hospital, TAIWAN

## Abstract

**Background:**

Acute stroke results in functional disability measurable using the well-known Barthel Index. The objectives of the study are to describe the change in the Barthel Index score and to model the prognostic factors for Barthel Index change from discharge up to 3 months post-discharge using the random intercept model among patients with acute first ever stroke in Kelantan, Malaysia.

**Methods:**

A total 98 in-hospital first ever acute stroke patients were recruited, and their Barthel Index scores were measured at the time of discharge, at 1 month and 3 months post-discharge. The Barthel Index was scored through telephone interviews. We employed the random intercept model from linear mixed effect regression to model the change of Barthel Index scores during the three months intervals. The prognostic factors included in the model were acute stroke subtypes, age, sex and time of measurement (at discharge, at 1 month and at 3 month post-discharge).

**Results:**

The crude mean Barthel Index scores showed an increased trend. The crude mean Barthel Index at the time of discharge, at 1-month post-discharge and 3 months post-discharge were 35.1 (SD = 39.4), 64.4 (SD = 39.5) and 68.8 (SD = 38.9) respectively. Over the same period, the adjusted mean Barthel Index scores estimated from the linear mixed effect model increased from 39.6 to 66.9 to 73.2. The adjusted mean Barthel Index scores decreased as the age increased, and haemorrhagic stroke patients had lower adjusted mean Barthel Index scores compared to the ischaemic stroke patients.

**Conclusion:**

Overall, the crude and adjusted mean Barthel Index scores increase from the time of discharge up to 3-month post-discharge among acute stroke patients. Time after discharge, age and stroke subtypes are the significant prognostic factors for Barthel Index score changes over the period of 3 months.

## Introduction

Acute stroke results in neurological, functional and cognitive disability [1–4]. The disability which affects the basic activities of daily living (ADL) post stroke can be measured using the Katz index of ADL, the functional independence measure and the Barthel Index. The Barthel Index has become the most commonly used outcome measure for disability [5], and is useful especially as a simple index in scoring improvement in rehabilitation. It is a scale that describes ten tasks and is scored according to amount of time or assistance required by the patient (index of independence) [6] and has excellent reliability [7] in an adult rehabilitation setting [8]. In contemporary stroke trials, the Barthel Index is the second most frequently used functional outcome measure behind the Modified Rankin scale [6, 9].

Studies on recovery after acute stroke have shown that the most useful prognostic factors are sex [10–12], age [13–17], stroke severity [18, 19], size of the stroke [14, 20, 21] and stroke subtype [22–24]. However, a drawback of previous studies has been is that they employed a cross-sectional study design or assumed independence or non-correlated errors in a longitudinal study design–which is not always valid. Serial data to examine disease or outcome trajectories should be analysed using specific methods such as linear mixed effect models [4, 25], which is the method used in this article.

In this study, we have the following research questions: a) how do Barthel Index scores change from the time of discharge to 1-month and 3-months post-discharge? b) Do time after discharge, age, sex and stroke affect the change of Barthel Index scores after discharge?

The aims of the study are, firstly: to describe the Barthel Index scores at the time of discharge, at 1-month and 3-month post-discharge and secondly; to model the prognostic factors (time after discharge, age, sex and stroke subtypes) for change in the Barthel Index score over a period of 3 months in acute first-ever acute stroke patients.

## Methods

### Patients

This was a retrospective cohort study, conducted between 1 July 2013 and 31 October 2014. We recruited 108 consecutive acute first ever stroke patients who were diagnosed and admitted in two major tertiary hospitals in Kelantan, Malaysia: a) Hospital Universiti Sains Malaysia (HUSM) and b) Hospital Raja Perempuan Zainab II (HRPZ) during the study period.

**Fig 1:**
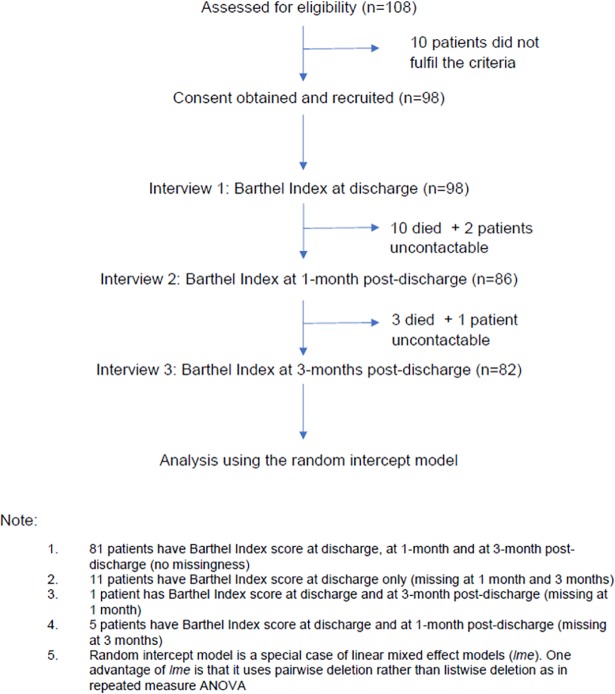
Study sample. 108 patients were assessed for eligibility. Ten patients did not fulfil the study criteria and ten died between discharge and one month post discharge. Three more patients died between discharge and three months.

From 108 patients, 98 patients were eligible for the study after they met the following inclusion criteria: a) if acute stroke was the primary diagnosis for hospital admission, b) age was > 18 years, c) if the acute stroke diagnosis was established by the neurology team, d) they were diagnosed and admitted between 1 July 2013 and 31 October 2014 and e) the acute stroke subtype was limited to: i) ischaemic stroke (infarction of the central nervous system) or ii) haemorrhagic stroke (spontaneous non-traumatic haemorrhage) [26]. The exclusion criteria were non-Malaysian nationality, diagnosis of recurrent stroke or failure to give the consent for the study. The rest, (n = 10 patients) were excluded because they did not have definitive diagnosis of either ischaemic or haemorrhagic stroke (see Fig 1).

### Barthel Index

The Barthel Index is a scale that indicates the ability to perform a selection of activities of daily living. It comprises 10 items (tasks), with total scores ranging from 0 (worst mobility in activities of daily living) to 100 (full mobility in activities of daily living) [6] and it has adequate clinimetric (quality of clinical measurements) properties in stroke rehabilitation [26]. In the index, the 10 items have these scoring combinations: a) 0 and 5, b) 0, 5 and 10, or c) 0, 5, 10 and 15. These items in the Barthel Index address a patient’s ability in feeding, bathing, grooming, dressing, bowel and bladder control, toileting, chair transfer, ambulation and stair climbing [27].

We treated the Barthel Index scale as a numerical scale rather than categorising it–because of the lack of consistency in selecting appropriate cut-off points for Barthel Index and reduction in information as a result of categorization [28–31].

### Interview

Each patient was interviewed three time: at discharge, at 1-month post discharge and at 3-month post-discharge (see Fig 1). At the time of discharge, the patient was still in the hospital and Barthel Index scores were elicited in the ward by visual inspection and face-to-face interview by the physician in-charge. At 1-month and 3-month post-discharge, the Barthel Index was scored using telephone-assisted interviews by a trained stroke nurse. A telephone-assisted interview for Barthel Index has these advantages: a) it is reliable and valid [32, 33], b) it can be used for proxies -such as carers- and lay persons [34–36]. A study has shown that there is no comparative difference between the Barthel Index administered via face to face versus via telephone interview [19]. Eliciting information from a proxy (usually the next of kin) in stroke studies is accurate [37] and has been used in the INTERSTROKE study (a large stroke study) [38].

### Prognostic factors

There were four prognostic factors in this study: time after discharge (time), age, sex and acute stroke subtypes.

There were three measurements of Barthel Index: a) at discharge, b) at 1-month post-discharge and c) at 3-month post-discharge. We chose 3 months as the maximum length of follow-up because the most significant recovery from neurological deficits after a stroke occurs during the first 3 months or 10 weeks [39, 40].

Neither hospital from which we recruited patients in our study had electronic medical record facility. Data for prognostic factors (sex, age and stroke subtypes) were abstracted by hand from the medical records by KIM. Stroke subtypes were recorded by a radiologist as either acute ischaemic or acute haemorrhagic stroke, age was recorded as the age of admission and sex as either male or female. Possible important prognostic factors such as depression, medical comorbidities and stroke location were not abstracted due to a significant number of missing information.

### Statistical analysis

Our study sample comprised 98 patients. All patients had the Barthel Index score at discharge. Between discharge and 1-month post discharge 10 patients died and 2 patients were uncontactable. Three more patients died between 1-month post-discharge and 3-month post discharge (see Fig 1). Eleven patients had missing Barthel Index score both at 1 month and 3 months post-discharge, one had missing score at 1 month only and 5 patients had missing score at 3 months only. And at 3 months, there were 16 patients with missing scores because 13 died and 3 patients were uncontactable. Hence, there were Barthel Index scores from 98, 86 and 82 patients, at discharge, 1-month post-discharge and 3-month post-discharge, respectively (Fig 1). Three patients had a mixed ischaemic and haemorrhagic stroke in the diagnosis and were labelled as missing stroke subtype.

The Barthel Index scores, age, sex and stroke subtypes were described using mean and SD for numerical variables or frequency and percentages (%) for categorical variables. Because the values of Barthel Index were different each time they were measured (at discharge, at 1-month and 3-month post-discharge), data of this kind (longitudinal data) require a method of analysis that takes account of the correlation between repeated measurements on the same patient. We used a linear mixed effect model (*lme*) [25, 41], specifically a random intercept model implemented with the ‘*lme’* function in the ‘*nlme’* package in R software [41], to analyse data from the 98 patients (with at least Barthel Index at discharge) who survived for at least 1 month post-stroke. Models with the main effect variables and 2-way interaction terms for the mean Bartel index were compared using the likelihood ratio (LR) tests. The level of statistical significance was set at p-value <0.05.

### Ethical approval and informed consent

This study received ethical approval from i) the Medical and Research Ethics Committee (MREC), Ministry of Health, Malaysia (NMRR-12-471-12139), ii) the USM Human Research Ethics Committee (HREC), Universiti Sains Malaysia (JEPEM [242.4.(1.4)]) and iii) the Lancaster University Research Ethics Committee.

We obtained written informed consent from all patients for this study. The capacity to consent was determined by the physician in-charge. In situation where the patients were deemed unfit by the physician, to provide written informed consent, the A carer who was the first-degree relative could provide consent on behalf of the patient.

## Results

The mean age of patients (n = 98) at baseline was 60.7 years. There were 34.7% (34/98) male and 65.3% (64/98) female patients. The female patients were slightly older (61.2 years) than the male patients (59.8). Ischaemic stroke patients comprised of 73.7% (70/95) of all patients in this study.

Table 1 shows that during the follow-up period, 13 patients died. Of those who died, 10 did so between the time of discharge and 1-month post-discharge, and 3 patients died between 1-month and 3-month post-discharge.

**Table 1:** Characteristics of study participants.

Characteristics	Types	n	Male, n(%)	Female, n(%)	All, n(%)
**Stroke subtype**	IS ^a^	70	27 (81.8)	43 (69.4)	70 (73.7)
	HS ^b^	25	6 (18.2)	19 (30.6)	25 (26.3)
**Mean Age (SD)**	Years	98	59.8 (12.6)	61.2 (14.2)	60.7 (13.6)
**At discharge**	Alive	98	34 (100.0)	64 (100.0)	98 (100)
**At 1 month after discharge**	Alive	88	28 (82.3)	60 (93.7)	88 (89.7)
	Dead	10	6 (17.7)	4 (6.3)	10 (10.3)
**At 3 months after discharge**	Alive	83	27 (79.4)	56 (90.3)	83 (86.5)
	Dead	13	7 (20.6)	6 (9.7)	13 (13.5)

^a^ IS = Ischaemic Stroke

^b^ HS = Haemorrhagic Stroke

In Table 2, the crude mean Barthel Index scores increase from 35.1 (SD = 39.4), to 64.4 (SD = 39.5) at 1-month and to 71.5 (SD = 38.9) at 3-month post-discharge. The mean Barthel Index score is lower at the time of discharge in females (mean = 31.6) than in males (mean = 41.8). At 1-month post-discharge, the scores for the females and males are almost similar (mean = 63.9 vs 65.6). The mean Barthel Index score at the time of discharge for the haemorrhagic stroke patients is lower (mean = 15.0) than that for ischaemic stroke patients (mean = 41.9). The scores then increase more than 4-folds between the time of discharge and 1-month post-discharge for both stroke subtypes.

**Table 2:** Barthel Index scores. The scores at discharge, at 1-month and at 3-month post-discharge.

Patients	n	At discharge	n	At 1 month	n	At 3 months
** **		Mean (SD)		Mean (SD)		Mean (SD)
**All**	98	35.1 (39.4)	86	64.4 (39.5)	82	71.5 (38.9)
**Male**	34	41.8 (42.4)	27	65.6 (41.3)	27	78.0 (34.6)
**Female**	64	31.6 (37.5)	59	63.9 (39.0)	55	68.3 (40.8)
**Ischaemic stroke**	70	41.9 (40.7)	61	63.0 (40.6)	57	70.3 (39.3)
**Haemorrhagic Stroke**	25	15.0 (27.2)	23	66.3 (38.1)	23	72.0 (39.6)

Table 3 shows four models (estimated from *lme* function in R) based on the combination of prognostic factors: measurement of time (Model 1), measurement of time + age (Model 2), measurement of time + stroke subtype (Model 3) and measurement of time + age + stroke subtype (Model 4). The adjusted Barthel Index scores at the time of discharge, at 1-month and 3-month post-discharge increase in trend (all models). Age is inversely related to the Barthel Index score in Model 2 and Model 3. The adjusted mean Barthel Index scores for haemorrhagic stroke are lower than for ischaemic stroke in Model 3 and Model 4.

**Table 3:** The estimated parameters. The four models (1 to 4) are estimated from linear mixed models.

Model	n	Covariates	Levels	Beta	SE	p-value
**1**	**98**	**Time**^**a**^	At discharge	35.10	3.95	<0.001
			1 month	62.44	4.09	<0.001
			3 months	68.82	4.14	<0.001
**2**	**98**	**Time + Age**	At discharge	35.11	3.81	<0.001
			1 month	62.37	3.96	<0.001
			3 months	68.77	4.00	<0.001
			Age	-0.80	0.25	0.002
**3**	**95**	**Time + Stroke subtype**^**b**^	At discharge	36.86	4.56	<0.001
			1 month	64.21	4.69	<0.001
			3 months	70.45	4.75	<0.001
			HS^c^	-7.66	8.20	0.353
**4**	**95**	**Time + Age + Stroke subtype**^**b**^	At discharge	39.64	4.44	<0.001
			1 month	66.89	4.57	<0.001
			3 months	73.18	4.62	<0.001
			Age	-0.94	0.27	0.001
			HS^c^	-16.77	8.22	0.044

^a^ Time is treated as dummy variables (at discharge, at 1-month post-discharge and at 3-month post-discharge

^b^ Stroke subtype = Haemorrhagic stroke (HS) vs Ischaemic Stroke (IS)

^c^ HS = Haemorrhagic stroke

Table 4 also shows the results of the fitting model with interaction terms. In Model 5, the covariates are the measurement of time + age + measurement of time × age. Model 6 on the other hand, contains the measurement of time + age + stroke subtype + age × stroke subtypes. Based on the LR test, adding the interaction terms (Model 5 and Model 6) have failed to significantly improve Model 4 (p = 0.123 and 0.535, respectively) further.

**Table 4:** The linear mixed models with the interaction term as the covariate. The p-values are >0.050 which support the exclusion of the interaction term from Model 5 and Model 6.

Model	n	Covariates	Variable	Beta	SE	p-value ^a^
**5**	**98**	**time**^**b**^ **+ age +**	At discharge	35.11	3.83	0.123
		**age×time**	1 month	62.31	3.97	
			3 months	68.69	4.02	
			age	-0.72	0.28	
			1 month × age	-0.13	0.25	
			3 months × age	-0.15	0.25	
**6**	**95**	**time**^**b**^ **+ age + stroke subtype +**	At discharge	39.90	4.43	0.535
		**age×stroke subtype**	1 month	67.16	4.56	
			3 months	73.46	4.61	
			HS^c^	-14.16	8.93	
			Age	-1.03	0.30	
		** **	Age × HS^c^	0.51	0.71	

^a^ p-values are 0.123 and 0.535, respectively. They are obtained from comparing the log-likelihood of the current model and Model 4)

^b^ time is treated as a dummy variable (at discharge, at 1-month after discharge and at 3 months’ after discharge)

^c^ HS = Haemorrhagic stroke vs Ischaemic stroke (reference).

**Fig 2:**
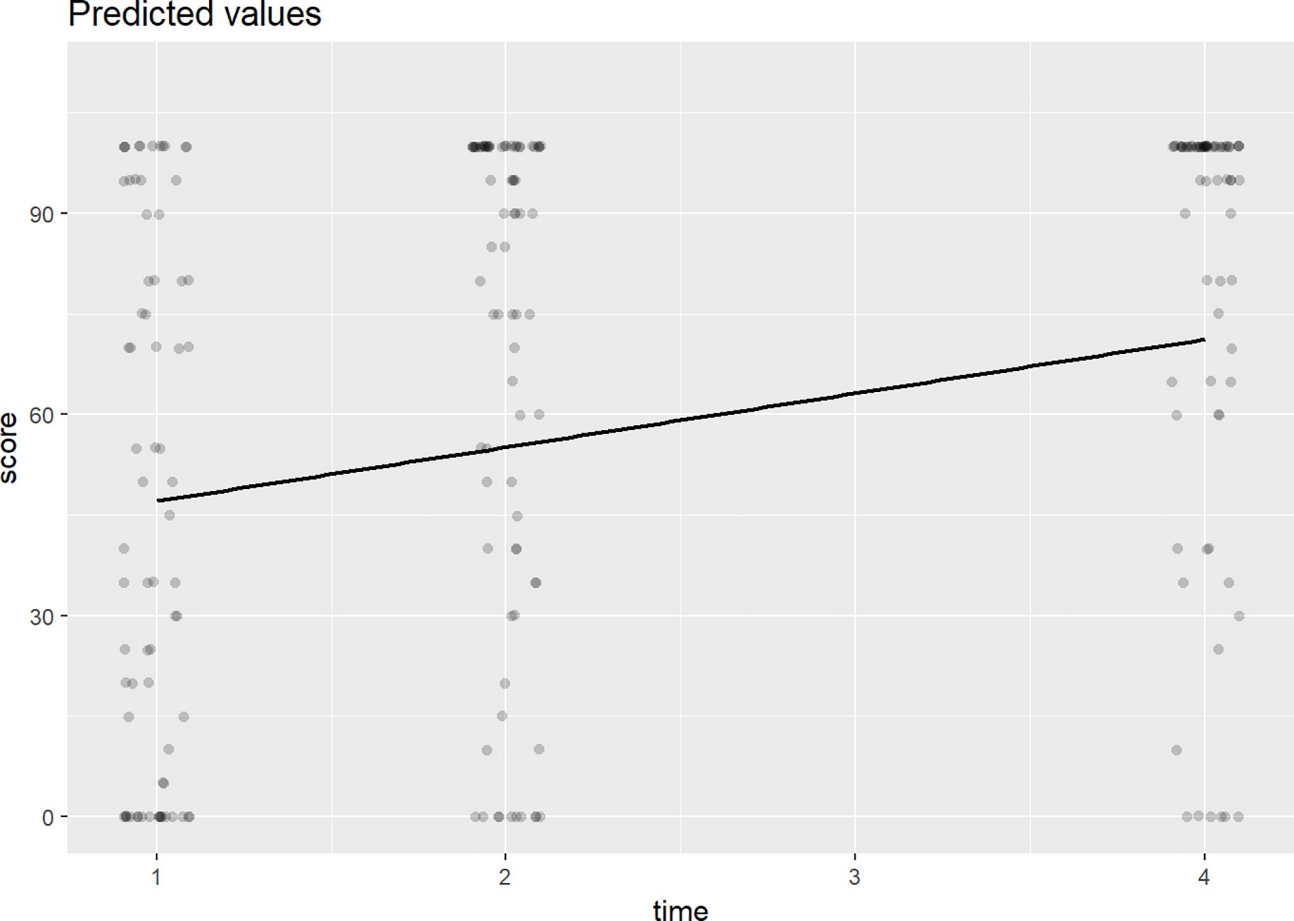
The predicted Barthel Index scores. These are the predicted scores for all subjects at discharge (time = 1), at 1-month after discharge (time = 2) and at 3-month after discharge (time = 4).

Fig 2 shows the fitted Barthel Index (the straight line) score for the three months’ interval estimated from Model 4 (covariates are time, age and stroke subtypes). Overall, the scores show an overall increase in the functionality based on the Barthel Index over time. The first Barthel Index score was assessed at discharge (time = 1). The second score was assessed at 1 month after discharge (time = 2) and the final score was taken at 3 months after discharge (time = 4). No Barthel Index score assessment is performed at 2 months after the baseline.

**Fig 3:**
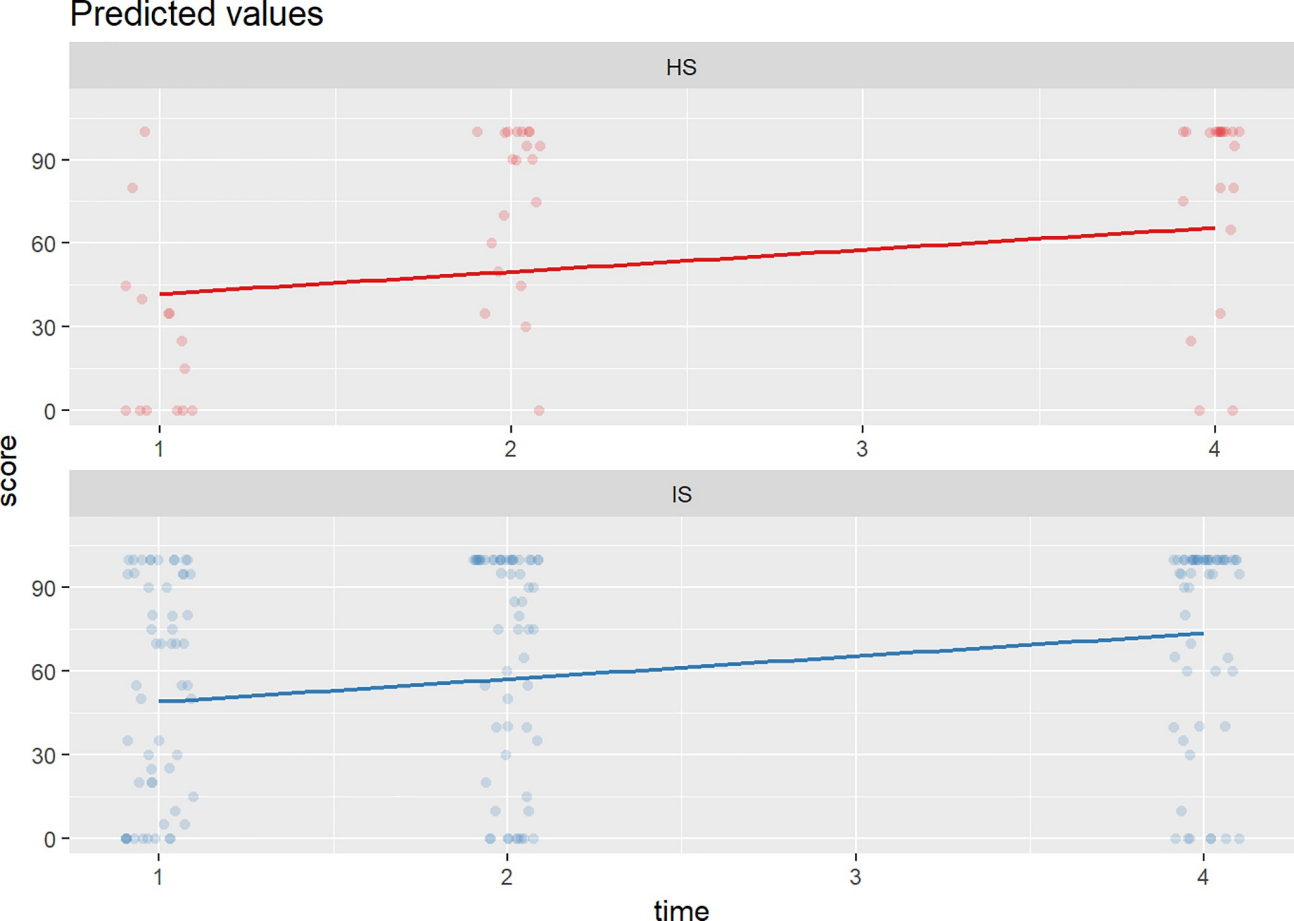
The predicted Barthel Index scores for stroke subtypes. These are the predicted scores for subjects with Haemorrhagic Stroke (HS) and Ischaemic Stroke (IS) at discharge (time = 1), at 1-month after discharge (time = 2) and at 3-month after discharge (time = 4).

Next, we obtained the fitted Barthel Index scores for patients Ischaemic Stroke (IS) and Haemorrhagic Stroke (HS) based on Model 4. The fitted Barthel Index scores for IS are consistently higher (better) than patients with HS (red line) (Fig 3). Both show an overall improvement in the Barthel Index over time.

## Discussion

This study has shown that in acute stroke patients, the crude Barthel Index scores increased from the time of discharge (mean = 35.1) to 1-month (mean = 64.4) and to 3-month post-discharge (mean = 71.5). Most recoveries took place between the baseline and 1 month after discharge. Measurements of time, age and stroke subtype are the essential prognostic factors for change in the Barthel Index scores.

In this study, the overall mean Barthel Index score increased by about 83.5% (from 35.1 to 64.4) and 104% (35.1 to 71.5) from the time of discharge to 1 month and from the time of discharge to 3 months respectively. It suggests that substantial recovery from acute stroke had taken place during these periods, especially between the time of discharge and 1 month. This early recovery can be attributed to the spontaneous neurological processes [42], and this occurs within 3 months after stroke [16]. Between 6 months and 2 years post-acute stroke, the Barthel Index score still improves but not considerably [43].

Our results showed that age was negatively associated with the Barthel Index. The inverse role of age on the Barthel Index is likely to be the result of various mechanisms which can be grouped into a) selective survival and/or cohort effect, b) physiologic are-related phenomenon, and c) increasing level of comorbidity due to ageing [44, 45]. It was likely that with the increasing age, the body becomes weaker, making recovery slower, brain tissue becomes damaged, and the protective effects of the endothelium and astrocytes in the brain are dysregulated [13] with a consequent negative effect on the sensory-motor recovery [46]. Our findings are in line with other studies that have shown age to be inversely associated with functionality [42, 47–49].

Our result support previous findings that acute stroke subtype plays a vital role in functional status [22, 23]. Haemorrhagic stroke was more severe due to more extensive brain injury as a result of a) the accumulation of blood and b) brain ischaemia following the haemorrhage [50]. Haemorrhagic stroke patients suffered more complications during in-hospital rehabilitation service [22], had a higher prevalence of mental disorders [51] and experienced poorer cognitive status [42] which rendeed them more vulnerable to slower recovery.

The strengths of our study include the fact that our follow-up was up to 3 months after the stroke—an appropriate period for showing clinical changes in functional status—as it has been shown that most motor recovery in stroke is often completed within 10 weeks of stroke [40]. We also employed appropriate statistical methods–in particular, the mixed effect model in the analysis instead of ordinary least square. The latter can severely over or underestimate the variance of the regression parameters in longitudinal data, and analysis of variance methods are not feasible for longitudinal data analysis [25].

One limitation of our study is that we took only 3 measurements over the 3-month period. To better quantify the changes in the Barthel Index score after stroke, more measurements are recommended during the follow-up partly because the improvements are extended until 12 months post-stroke [16]. Secondly, the sample size should be larger to accommodate more covariates in the model. Thirdly, more data from other covariates such as psychological and employment variables are necessary to provide more valid results.

We also recognize that the inclusion criteria were wide which increases the heterogeneity of the study. We used telephone interview which has a a tendency to provide incomplete information, a higher level of missing data, considerable difficulty in achieving rapport and the lack of visual cues [52] perhaps due to aphasia or cognitive decline. This consequence of telephone interviews were minimized by interviewing the carers. Lastly, we did not include other indices such as Motricity Index (to measure strength of extremities after stroke) [53], Katz Index, Functional Ambulation Classification (FAC) [54], Walking Handicap Scale (WHS) [55], which could provide a more rounded representation of the functional and outcome assessments.

## Conclusions

The overall mean Barthel Index score increases consistently from the time of discharge to 1-month post-discharge and then to 3-month post-discharge, indicating functional recovery in acute stroke patients. The score largely improves between the time of discharge and at 1-month after discharge (68.7% improvement) compared to between 1 month and 3 months post-discharge (9.4% improvement). The prognostic factors for the change in Barthel Index after acute stroke include time, age and stroke subtypes.

## Supporting information

S1 File Supporting information. The assumptions for generalized mixed effect models and the results from generalized estimating equation (GEE).
(DOCX)
