# Supplementary material for: The change of Barthel Index scores from the time of discharge until 3-month post-discharge among acute stroke patients in Malaysia: A random intercept model
Source: PLoS One. 2018 Dec 20;13(12):e0208594. doi: 10.1371/journal.pone.0208594 (PMC6301695; doi:10.1371/journal.pone.0208594)
Supplement: S1 File — The assumptions for generalized mixed effect models and the results from generalized estimating equation (GEE). (DOCX) [file pone.0208594.s001.docx]

# **S1 File. Supporting information**

## Assumptions

We noticed that in the generalized linear mixed effect models that we performed,

- - - 1. the empirical correlation structure of the residuals does not conform to the assumed "random intercept" structure (under which the variance is the same at each of the three time-points and the covariance is positive and the same between any two time-points) and
      2. the distribution of the response variable is grossly non-normal.

Due to these, we run a generalized estimating equations (GEE) based on the recommendation by Liang, K-Y and Zeger, S.L [1] to examine the discrepancy between the linear mixed effect model and GEE. The results are very similar (see S1 Table).

**S1 Table.** Estimation based on generalized estimating equation

| **Variables** | **Beta** | **Robust SE** | **Robust Z** | **p-value** |
| --- | --- | --- | --- | --- |
| **At discharge** | 66.85 | 4.49 | 14.87 | <0.001 |
| **At 1-month** | 73.13 | 4.38 | 16.71 | <0.001 |
| **At 3-month** | 39.64 | 4.52 | 8.78 | <0.001 |
| **Age** | -0.95 | 0.25 | -3.84 | <0.001 |
| **^C^ HS** | -16.79 | 6.99 | -2.40 | 0.016 |

^c^ HS=Haemorrhagic stroke vs ischaemic stroke

## Reference

1. Liang K-Y, Zeger SL. Longitudinal data analysis using generalized linear models. Biometrika. 1986;73(1):13-22. doi: 10.1093/biomet/73.1.13.
